# Virtual and real assessment of a wide antral ablated region in atrial fibrillation patients using the hot balloon system

**DOI:** 10.1002/ccr3.3730

**Published:** 2021-01-05

**Authors:** Reiko Fukuda, Shiro Nakahara, Hirotsugu Sato, Naoki Nishiyama, Yuichi Hori, Isao Taguchi

**Affiliations:** ^1^ Department of Cardiology Dokkyo Medical University Saitama Medical Center Saitama Japan

**Keywords:** atrial fibrillation, catheter ablation, computer simulation, hot balloon ablation

## Abstract

The hot balloon system has become widely used for atrial fibrillation ablation and also has software for preoperative computer simulation. The computer model may be useful for predicting the extent of a wide planar ablation region in the left atrium.

## Graphical abstract

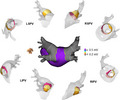

## 1 INTRODUCTION

Despite significant innovations in balloon‐based ablation of atrial fibrillation (AF), no attempts of preoperative simulation with a computer model have been performed. We present a case of paroxysmal AF using the hot balloon system in which a post hoc simulation of the balloon contact region by a computer‐aided engineering analysis was performed.

Balloon technologies are known to be an effective therapy for atrial fibrillation (AF). Hot balloon ablation is performed with a compliant balloon that maximally contacts irregularly shaped PV antra.^1^, ^2^ One can assume that hot balloon systems would create large and durable planar ablation lesions including wide antral regions.^3^ However, despite significant innovations in balloon‐based ablation over the past decade, no attempts of preoperative simulation with a computer model have been performed. We present a case with a virtual assessment of a wide antral ablated region in AF patients using the hot balloon system.

## 2 CASE DESCRIPTION

**FIGURE 1:**
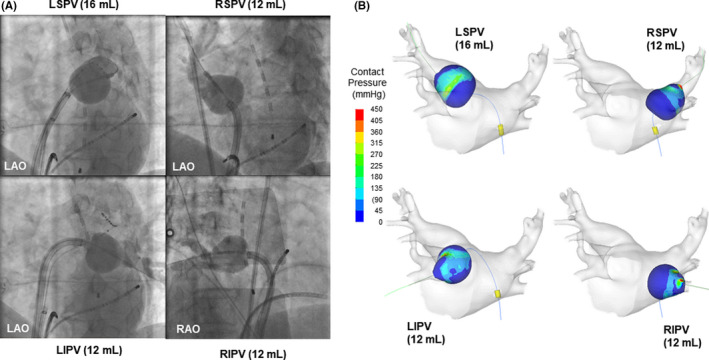
A. Periprocedural fluoroscopy. B. CAE model of the hot balloon. The estimated balloon contact with the tissue of each PV antral area of > 45mmHg is indicated in light blue

A 68‐year‐old male patient underwent a pulmonary vein isolation (PVI) for paroxysmal AF using a radiofrequency hot balloon catheter (SATAKE HotBalloon; Toray Industries, Tokyo, Japan). A single energy application with an injected volume of 12‐16 mL of contrast medium diluted 1:2 with saline was performed for each of the four PV ostia (not inside the PV) (Figure 1). A successful PVI was achieved for all four PVs, and high‐density mapping was performed. No AF recurrence was documented 1 year later.

**FIGURE 2:**
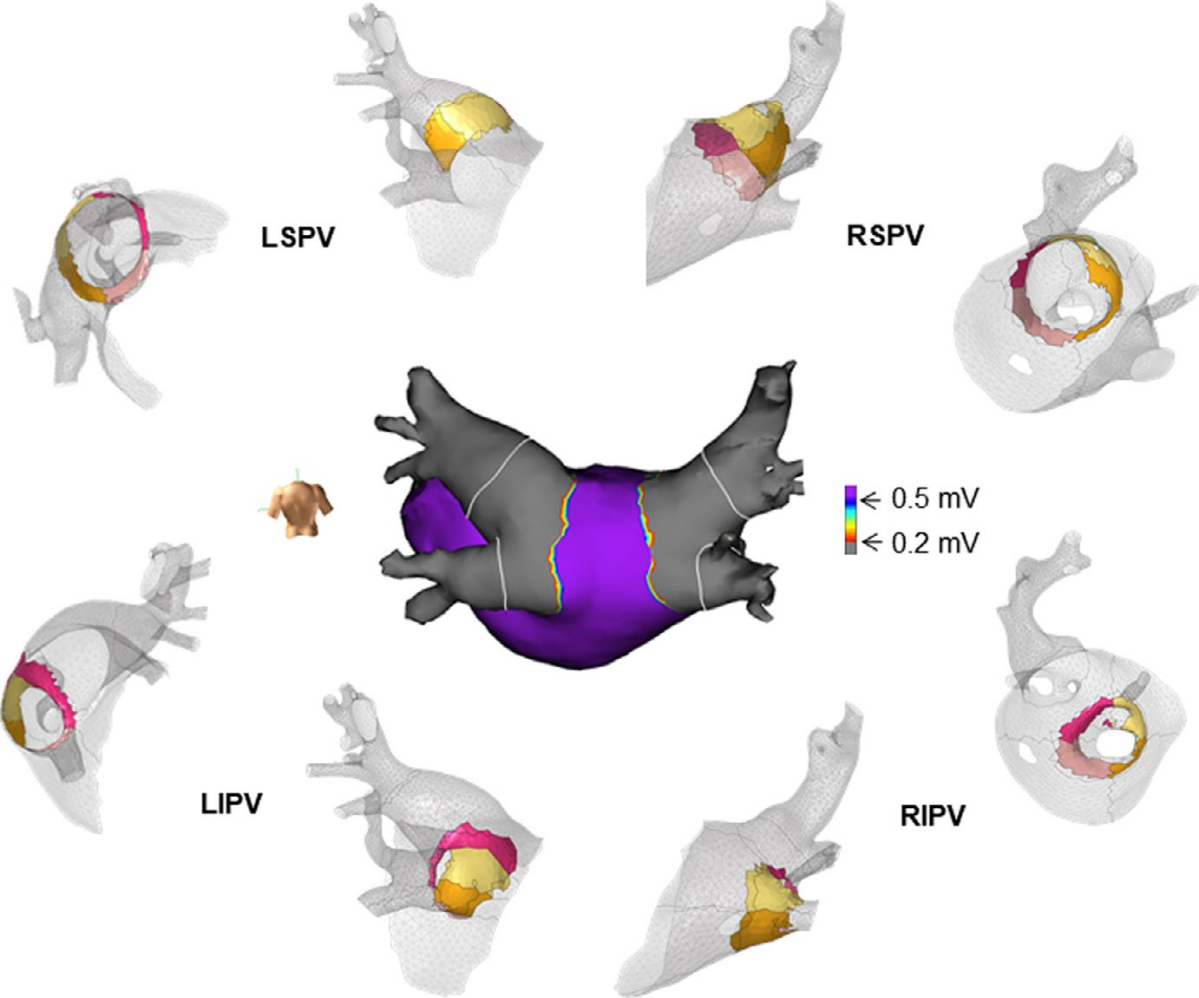
The quantified balloon contact area in the CAE model and intraoperatively recorded high‐density voltage map of the LA

After the procedure, an offline virtual simulation to evaluate the actual balloon contact area with the atrial tissue was demonstrated. The analysis was performed using computer simulation technology called computer‐aided engineering (CAE) to continuously visualize the contact area between the hot balloon and PV antrum. The patient's 3D‐CT DICOM data were incorporated into the heart with the SATAKE HotBalloon model for the CAE using LS‐DYNA software (LSTC Inc, Livermore Calif). The position of the transseptal puncture was also incorporated into the system with reference to the cine images. After placing the hot balloon model at the appropriate position in the heart model, the plain contact of the hot balloon surface to the cardiac tissue was virtually assessed using this system (Figure 2; see the Supplemental Movie S1). The balloon injection volume for each PV in this model was the same as the use in the actual clinical setting. As the figure demonstrates, a seamless planner balloon contact was virtually confirmed at each balloon location. Furthermore, the quantified contact area determined by the CAE model correlated with the low voltage region (<0.2 mV) around the PV antrum determined by the high‐density voltage map (CAE vs. voltage map; left side; 20.4 cm^2^ vs 19.7 cm^2^ , right side; 14.8 cm^2^ vs 14.9 cm^2^; Figure 2).

## 3 DISCUSSION

CAE means computer‐aided engineering, and it is capable of simulating a wide variety of physical phenomena in both the industrial world and the biomedical engineering.^4^ In this analysis, real parts were expressed as a divided mesh division. By setting each parameter for each mesh, it can simulate various phenomena by using mechanical equations. In our study, we used a specific program of the SATAKE HotBalloon and human heart in conjunction with patient's CT DICOM data. Furthermore, the CAE model successfully replicated the atrial seamless planar balloon contact with the targeted tissue. To the best of our knowledge, this is the first report of the simulation of balloon‐based ablation for AF.

Further prospective study is warranted to determine whether a CAE analysis is useful for a preoperative simulation to predict an appropriate injection dose for the hot balloon in each patient.

## 4 CONCLUSIONS

This case demonstrated the novel and potential value of a CAE analysis for a preoperative simulation of hot balloon ablation of AF. The CAE model may be useful for predicting both the recognition of the position of the hot balloon and the extent of the wide planar ablation region in the left atrium.

## CONFLICT OF INTEREST

Nothing to declare.

## AUTHOR CONTRIBUTIONS

RF, SN, and HS: performed the procedure and prepared the manuscript. NN, YH, and IT: critically revised the manuscript.

## ETHICAL APPROVAL

All ethical concerns were respected throughout the elaboration of this case report.

## Supporting information

Video S1

Video S2

Supplementary Material
