# Supplementary figures and images for: Virtual and real assessment of a wide antral ablated region in atrial fibrillation patients using the hot balloon system
Source: Clin Case Rep. 2021 Jan 5;9(3):1199–201. doi: 10.1002/ccr3.3730 (PMC7981672; doi:10.1002/ccr3.3730)

## Slide 1
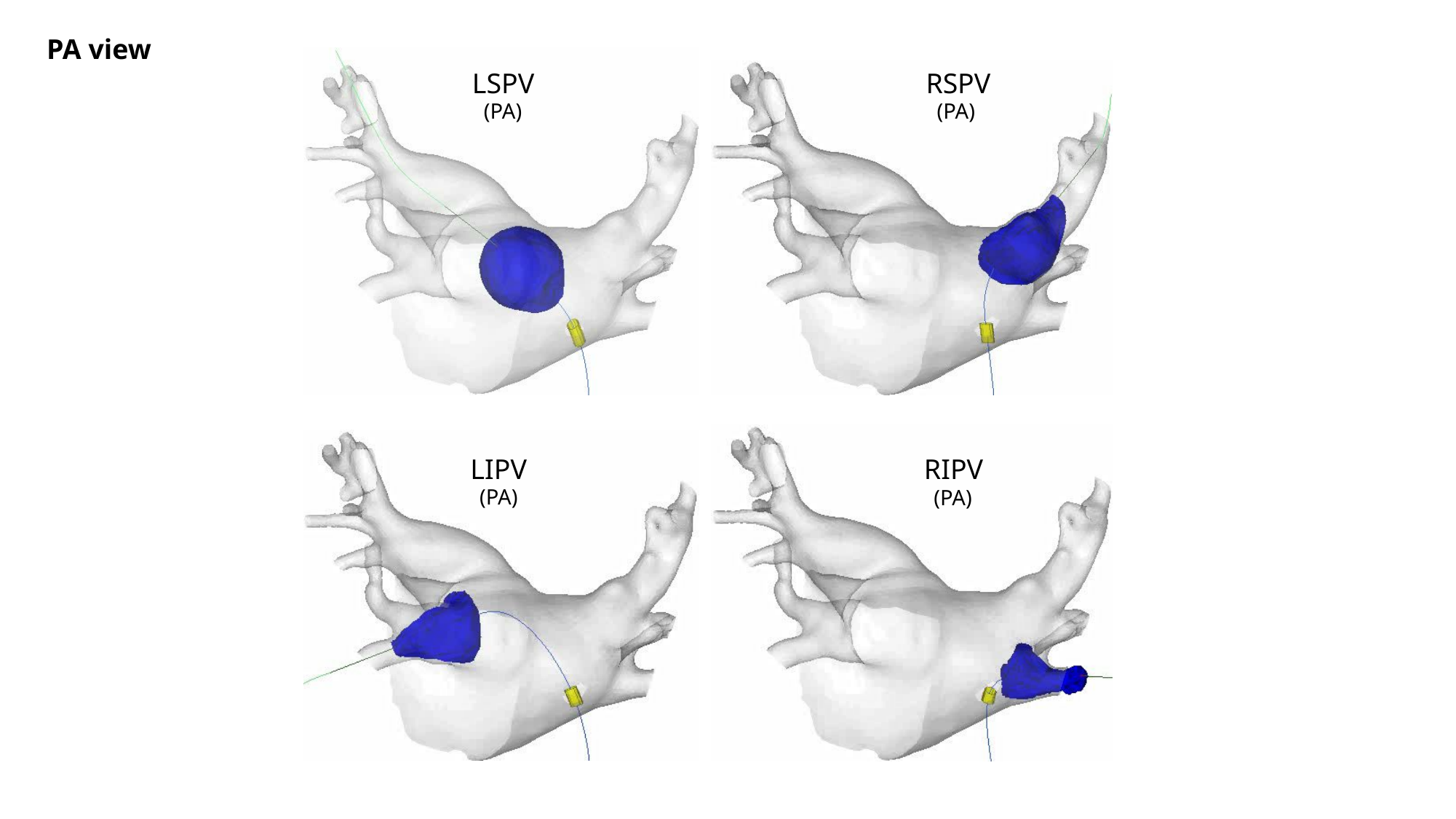

PA view
LSPV
RSPV
(PA)
(PA)
LIPV
RIPV
(PA)
(PA)

Supplement: Supplementary file 1 — Video S1 [file CCR3-9-1199-s003.pptx]
