# Supplementary material for: Virtual and real assessment of a wide antral ablated region in atrial fibrillation patients using the hot balloon system
Source: Clin Case Rep. 2021 Jan 5;9(3):1199–201. doi: 10.1002/ccr3.3730 (PMC7981672; doi:10.1002/ccr3.3730)
Supplement: Supplementary file 3 — Supplementary Material [file CCR3-9-1199-s001.docx]

**Supplementary Movies**

Actual demonstration of the CAE model from multiple views (1 movie).
